# Ablation catheter–induced mechanical deformation in myocardium: computer modeling and ex vivo experiments

**DOI:** 10.1007/s11517-024-03135-7

**Published:** 2024-06-01

**Authors:** Yukako Ijima, Kriengsak Masnok, Juan J. Perez, Ana González-Suárez, Enrique Berjano, Nobuo Watanabe

**Affiliations:** 1 Biofluid Science and Engineering Laboratory, Global Course of Engineering and Science, Graduate School of Engineering and Science, Shibaura Institute of Technology, 307 Fukasaku, Minuma-Ku, Saitama-City, Saitama, 337-8570 Japan; 2 Department of Industrial Engineering, Faculty of Engineering, Srinakharinwirot University, Ongkharak, Nakhon Nayok, Thailand; 3 BioMIT, Department of Electronic Engineering, Universitat Politecnica de Valencia, Camino de Vera, 46022 Valencia, Spain; 4 Translational Medical Device Lab, School of Medicine, University of Galway, Galway, Ireland; 5 Universidad Internacional de Valencia, Valencia, Spain; 6 Biomedical Engineering Course, Dept. of Bio-Science and Engineering, College of Systems Engineering and Science, Shibaura Institute of Technology, 307 Fukasaku, Minuma-Ku, Saitama-City, Saitama, 337-8570 Japan

**Keywords:** Cardiac ablation, Cardiac catheter, Computer modeling, Contact force, Mechanical deformation, Mechanical model

## Abstract

Cardiac catheter ablation requires an adequate contact between myocardium and catheter tip. Our aim was to quantify the relationship between the contact force (CF) and the resulting mechanical deformation induced by the catheter tip using an ex vivo model and computational modeling. The catheter tip was inserted perpendicularly into porcine heart samples. CF values ranged from 10 to 80 g. The computer model was built to simulate the same experimental conditions, and it considered a 3-parameter Mooney-Rivlin model based on hyper-elastic material. We found a strong correlation between the CF and insertion depth (ID) (*R*^2^ = 0.96, *P* < 0.001), from 0.7 ± 0.3 mm at 10 g to 6.9 ± 0.1 mm at 80 g. Since the surface deformation was asymmetrical, two transversal diameters (minor and major) were identified. Both diameters were strongly correlated with CF (*R*^2^ ≥ 0.95), from 4.0 ± 0.4 mm at 20 g to 10.3 ± 0.0 mm at 80 g (minor), and from 6.4 ± 0.7 mm at 20 g to 16.7 ± 0.1 mm at 80 g (major). An optimal fit between computer and experimental results was achieved, with a prediction error of 0.74 and 0.86 mm for insertion depth and mean surface diameter, respectively.

**Graphical Abstract:**

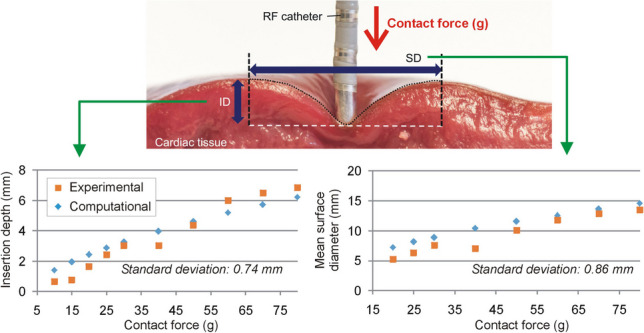

**Supplementary Information:**

The online version contains supplementary material available at 10.1007/s11517-024-03135-7.

## Introduction

Cardiac arrhythmia, or an abnormal heartbeat, is one of the most common diseases that can happen at any age [1]. For example, only in Japan, it is estimated that 4% of men and more than 2% of women in their 80 s have this disease, while the number of patients was around 1 million in 2020 but is expected to increase in the future due to population aging [2]. The only definitive method for treating cardiac arrhythmia is cardiac ablation, which can be made minimally invasive by using an ablation catheter. Different energy sources can be used to ablate the tissue in the target zone, such as radiofrequency (RF), microwave, or high voltage pulses (pulsed field ablation). In the case of RF catheter ablation (RFCA), RF current is applied to the zone identified as the origin of the abnormal heart rhythm by means of an active metal electrode on the catheter tip, which creates a thermal lesion by the Joule effect in the cardiac tissue just below the electrode [3]. In the initial phase of the RF power conversion process, resistive heating starts when the tissue zone receives a very high current, which occurs in quite a small tissue volume within 1 − 2 mm from the electrode-tissue interface. The heat is then transferred through thermal conduction from the initial high-temperature zone to the lower-temperature zones in the deeper myocardial layer [4–6]. The thermal lesion is formed at lethal temperatures (~ 50 − 55 °C) for several seconds, and the tissue is irreversibly damaged by coagulative necrosis [7, 8].

The active electrode requires adequate contact with the cardiac tissue surface to generate an effective thermal lesion, i.e., a sufficiently large electrode-tissue contact area. Our previous experimental studies showed that the catheter contact force (CF), which is automatically measured by most commercially available ablation catheters, is closely related to the electrode-tissue contact area for any catheter position [9] and that while CF is strongly correlated to the RF-induced lesion area, the correlation is even stronger between electrode-tissue contact area and lesion area [10]. It can thus be concluded that the electrode-tissue contact area is a critical parameter in predicting the size of the thermal lesion, since it controls how much RF power will be effectively focused on the myocardium and how much RF power will be “lost” in the blood pool. In fact, in certain conditions such as when the electrode is inserted deep into a trabeculated area, very different lesions can be created with similar CF values, while the electrode-tissue contact area (also called the coverage level between the electrode and cardiac tissue) significantly affects the lesion size [11]. In addition, the surface deformation could also play a very important role in terms of conditioning the map of blood and saline flow velocities irrigated in the surroundings of the electrode.

In this respect, as cardiac muscle is known to be elastic and liable to deformation, the electrode-tissue contact area will be the result of mechanical deformation both in depth and in width. To our knowledge, only two studies have assessed the mechanical deformation caused by an ablation catheter placed on the myocardium surface. Cao et al. [12] used fresh bovine hearts and X-ray images to characterize the deformation contour obtained for electrode penetration depths of from 0 to 6 mm. Although they quantified the electrode area covered by myocardium and the lateral extension of the deformation, the CF was not assessed. Choy et al. [13] measured the relationship between the CF and penetration depth of the ablation electrode into the myocardium but did not assess the tissue’s lateral deformation. No studies have therefore yet investigated the full relationship between CF and the resulting mechanical deformation in terms of insertion depth and surface deformation.

**Fig. 1:**
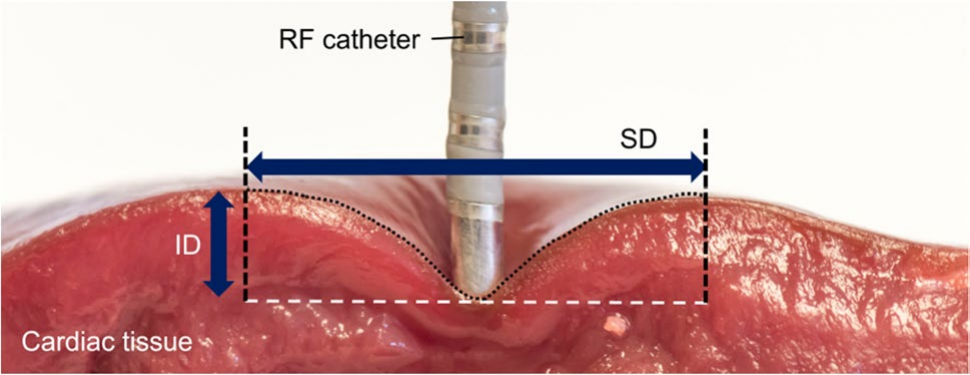
Side view of the deformed tissue (dotted black line) with an electrode placed on the surface of the cardiac tissue. The deformation was characterized by the ID (insertion depth measured from the tissue surface to the electrode tip) and SD (surface diameter of the deformed area)

Computer modeling has proven to be a very useful tool in the study of RFCA [3]. While most recent computer modeling studies have emphasized the importance of tissue surface deformation caused by the CF between electrode and tissue on the thermal lesion size [14–17], none has offered an experimental validation in terms of mechanical response, i.e. the surface deformation induced by the CF. Our objectives were thus (1) to experimentally quantify the relationship between the CF and the mechanical deformation of the tissue produced in an ex vivo porcine heart model and (2) to develop and validate a computational model able to predict the relationship between the CF and surface deformation, characterized by the insertion depth (ID) of the electrode inside the tissue and the surface diameter (SD) of the deformed tissue (see Fig. 1).

## Materials and methods

### Ex vivo porcine heart model

**Fig. 2:**
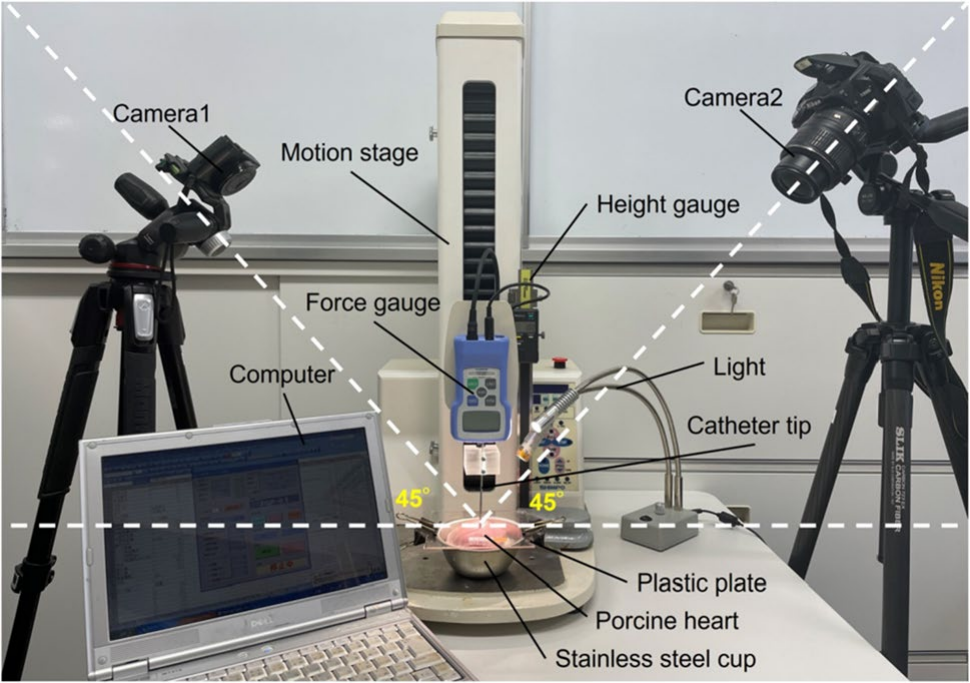
Overview of the experimental setup used to assess the deformation produced on the tissue surface by the catheter. White dashed lines represent camera angle in relation to the tissue surface (horizontal line)

Fresh porcine hearts were obtained from a commercial slaughterhouse on the day after the animals’ sacrifice. The hearts were washed in a saline solution (0.9 wt%), and then sections of both left and right ventricular myocardium were cut and placed in the recipient used in the experiments. All the sections were stored at room temperature in a saline solution until the experiment was started. Figure 2 gives an overview of the experimental setup. The entire ventricle was placed in a stainless steel container, and a transparent methacrylate plate was placed on the surface of the epicardium without adipose tissue to allow easy visualization of the deformed area with the catheter tip on the surface, especially the borders of the deformed area (see Fig. 1). A 2.33-mm diameter flat metal catheter tip (smooth edge blunt tip) was placed at the center of the force gauge. Different CF values were reached on the tissue surface by moving the catheter metal tip by a motion stage model FGS-5000TV (Nidec-Shimpo Corporation, Kyoto, Japan). The CF values were measured by means of a force gauge model FGP-0.5 (Nidec-Shimpo Corporation, Kyoto, Japan), which automatically stopped the motion stage once the CF target was reached. Ten CF levels were assayed three times: 10, 15, 20, 25, 30, 40, 50, 60, 70, and 80 g. So far, this has been the approximate typical range considered in experimental studies [9, 10]. The lower-range values (< 20 g) are of course used in clinical practice in areas where a more superficial lesion is desired, such as the posterior wall of the left atrium, while higher values can be applied in other areas such as the ventricular wall. The system was operated and monitored by FGT-TV software (Nidec-Shimpo Corporation, Kyoto, Japan) running on a laptop. A height gauge model HDS-C (Mitutoyo, Kawasaki, Japan) was used to measure the ID. Due to the force gauge being located on the top of the catheter, photography of the deformation area from the top view was not possible. Hence, in order to measure the diameter of the deformation area, two cameras were placed on the opposite side of the porcine heart at 45° angle to acquire two images from each side. The use of two cameras was required to capture the deformed area just behind the catheter metal tip and achieve in this way a 360° view of the deformed area (see Section 2.2).

### Image analysis

**Fig. 3:**
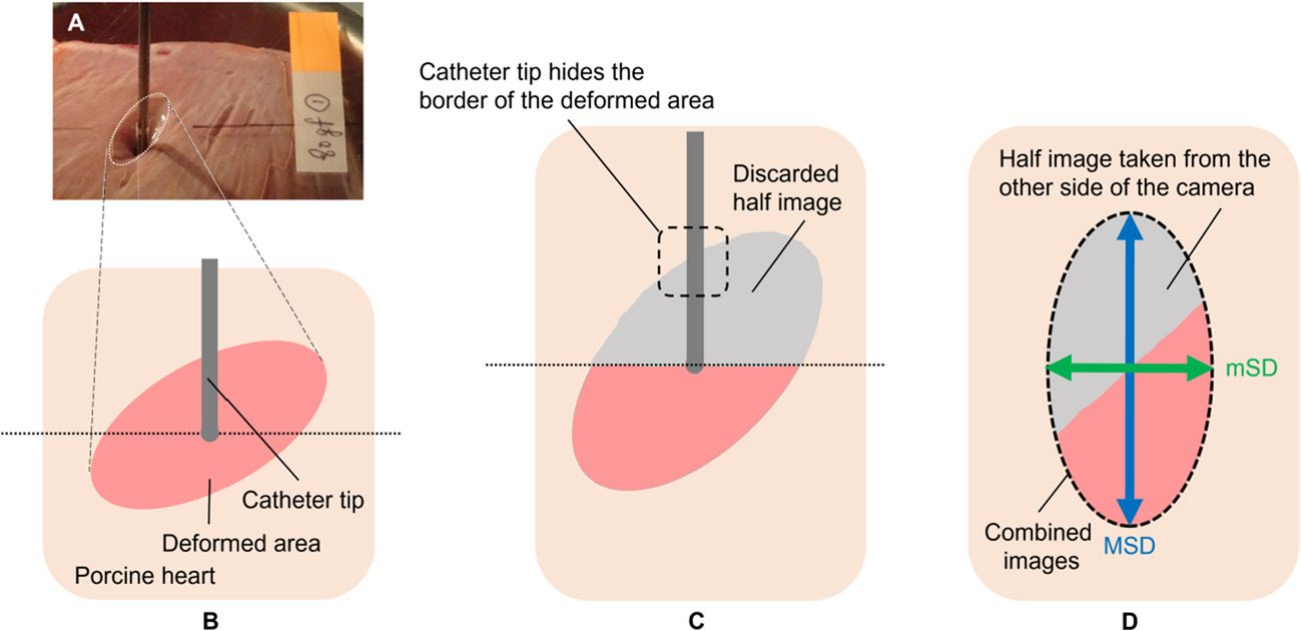
Procedure conducted to process the visual information from two cameras. **A** Real image from one camera (at 80 g CF). **B** The boundary of the deformed area was visually identified from each side. **C** The images were converted into the top view by MATLAB, and the half of the deformed zone just behind the catheter was discarded. **D** The images from both sides were combined to obtain the full visual description of the deformed area to measure the diameters of the surface deformation (major surface diameter MSD, and minor surface diameter mSD)

Figure 3 shows the procedure used to analyze the camera images. Two lamps were used to light the entire perimeter of the deformed surface area (see Fig. 1). First, each image was converted into the top view image by the MATLAB program (MathWorks, MA, USA). The border of the deformed area was quantified by ImageJ software (National Institutes of Health, MD, USA), discarding the half of the image just behind the catheter. The same process was repeated on the image taken from the other side. Images from both sides were combined to obtain the full visual description of the deformed area. As the preliminary results showed that these areas were not completely circular, we decided to measure two surface diameters: major (MSD) and minor (mSD).

### Statistics

The relationships between the outcomes (ID and SD of the deformed area) and the CF values were studied by simple regression. The coefficient of determination (*R*^2^) was reported to assess the goodness of fit. Statistical significance was assumed when the *P* value (*P*) was lower than 0.05.

### Computational model

**Fig. 4:**
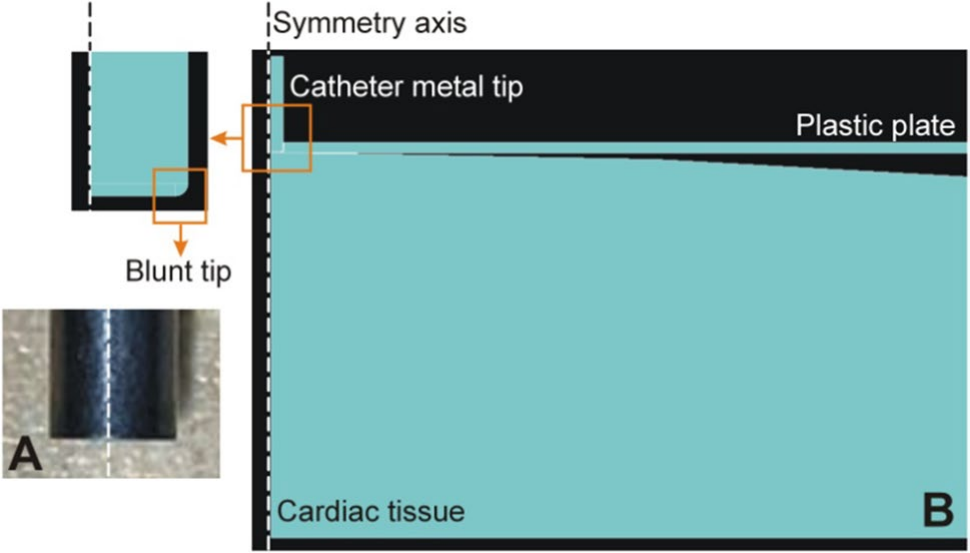
**A** Detail of the cylindrical catheter with a blunt metal tip. **B** Geometry of the axisymmetric model including a fragment of cardiac tissue, a metal catheter tip, and a plastic plate

We built a 3-parameter hyper-elastic Mooney-Rivlin computer model based on the Finite Element Method using ANSYS software (ANSYS, Canonsburg, PA, USA), to simulate conditions closely resembling those used in the experiments. Figure 4 shows the geometry of the 2D model with axial symmetry, which included a fragment of cardiac tissue, the catheter tip, and a plastic plate representing a 1-mm thick rectangular transparent methacrylate plate placed on the tissue to achieve a “flat surface” and to serve as a reference point from which to measure the insertion depth (see Section 2.1). This was important, since the compression exerted by the plate on the tissue surface has a mechanical impact on the result. The catheter tip was modeled as a stainless-steel 2.33 mm-diameter cylinder. To avoid mechanical tension in the electrode’s lower edges, the tip was modeled as blunt but with a fillet radius equal to 1/8th of the electrode radius (similar to the electrode curvature in the experiments, as shown in Fig. 4A). The electrode was 10-mm long (this value has an insignificant impact on the results, since the electrode is much harder than the other elements).

Generic values of the mechanical characteristics of a non-deformable element (Young’s modulus E = 1.7 × 10^11^ Pa and Poisson’s ratio Nu = 0.3) were chosen for the metal electrode and plastic plate. We assumed hyper-elastic material and a Mooney-Rivlin model for the cardiac tissue, with a strain energy density function given by the following:

1 $$W={c}_{10}\left({\overline{I}}_{1}-3\right)+{c}_{01}\left({\overline{I}}_{2}-3\right)+{c}_{11}\left({\overline{I}}_{1}-3\right)\left({\overline{I}}_{2}-3\right)+\frac{1}{d}{\left(J-1\right)}^{2}$$

where *W* is the potential energy of deformation; *I*1 and *I*2 are the first and second invariants of the unimodular component of the Cauchy-Green tensor; *c*_*10*_, *c*_*01*_, *c*_*11*_, and *d* are material constants; and *J* = *det*(*F*), where *F* is the deformation gradient. The relationship between *d*, c_*10*_, and *c*_*01*_ and Poisson’s ratio is as follows: If the latter is fixed at 0.49, assuming practical incompressibility [18], the parameter *d* is fixed by the others (i.e. it is not an unknown). The entire model (including all the subdomains) was meshed with 12,600 elements and 25,694 nodes. The grid size ranged from 27 μm at the electrode tip to 2.4 mm at the outer limits of the tissue. Both the mesh size and the tissue’s outer dimensions (8 cm radius, 4 cm high) were checked by means of a convergence test. For this, we conducted simulations by consecutively decreasing the mesh size and enlarging the outer dimensions until finding a difference of the insertion depth of less than 0.1 mm between consecutive simulations. We assumed a direct contact between the electrode and tissue surface, as well as between the lower surface of the plastic plate and the tissue surface. The coefficient of friction between any pair of surfaces was zero.

In the boundary condition, the horizontal displacement of the lines in the model’s symmetry axis was zero, as was the displacement of the plastic plate. We set a mechanical load at the bottom of the model consisting of an upward displacement, which followed a ramp starting from zero until reaching value *D* (in mm) after 0.1 s. Uniform downward pressure was then applied to the top of the electrode, with a value equivalent to the weight of the CF, following a ramp from 0 to the equivalent CF value from 0.1 to 1 s. The simulations were conducted in static mode, i.e., without considering inertial forces. The large deformation option was activated in ANSYS.

The CF was changed in the same range as in the experiments. ID and the average value between mSD and MSD were computed for each CF value (see Fig. 1). We calculated the mean square error (MSE) by assuming the relationship between CF and ID as follows for a specific set of values of *c*_*10*_,* c*_*01*_,* c*_*11*_, and* D* and the 10 CF values used in the experiments (i.e., 10, 15, 20, 25, 30, 40, 50, 60, 70, and 80 g):

2 $$MSE=\frac{1}{10}\sum\nolimits_{1=1}^{10}{\left({ID}_{Comp}-{ID}_{Exp}\right)}^{2}$$

where *ID*_*Com*_ is the insertion depth obtained from the computer simulations and *ID*_*Exp*_ is the mean value of ID obtained in the experiments so that MSE is hence a function of *c*_*10*_*, c*_*01*_,* c*_*11*_, and* D*. To find the data set that minimizes MSE and therefore achieve a better fit between the computer and experimental data, the gradient descent algorithm (also known as the steepest descent) was constructed from a certain probable data set, and MSE was calculated for this combination. The MSE gradient was calculated for this combination and moved forward in search of the minimum MSE in the opposite direction to the calculated gradient. The Barzilai-Borwein method was used to calculate the progress made in the opposite direction to the gradient [19].

## Results

### Experimental results

**Fig. 5:**
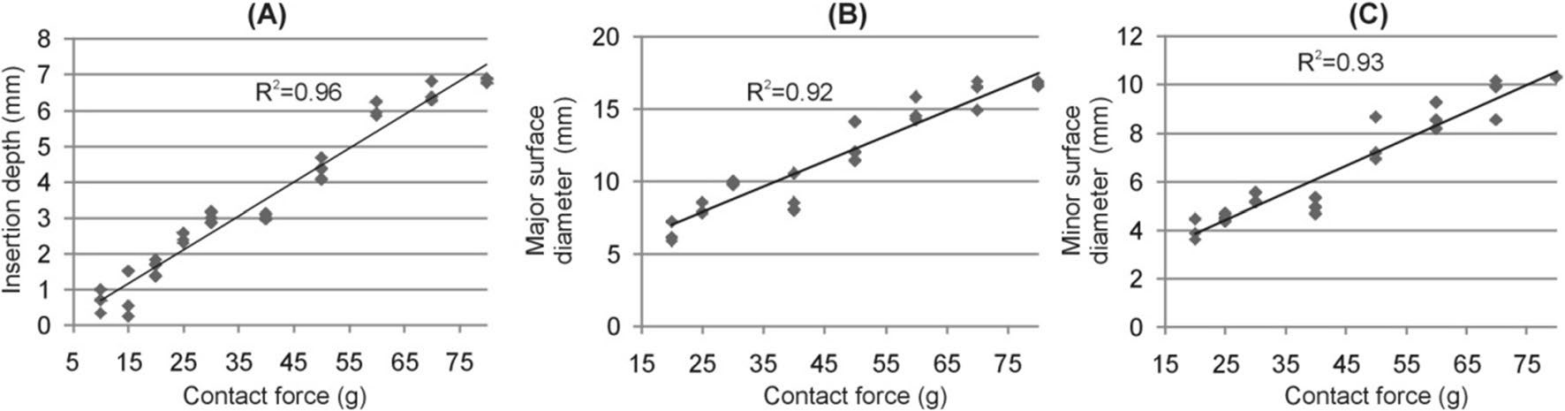
Relationship between contact force (CF) and insertion depth (**A**), major surface diameter (**B**), and minor surface diameter (**C**) of the deformed area produced (*n* = 3 for each CF value)

Figure 5 shows the relationships between the CF and the outcomes of the deformation produced (ID, MSD, and mSD). It was not possible to measure the CF surface diameters below 20 g because the deformed area was hardly visible. We found a strong positive correlation between ID and CF (*R*^2^ = 0.96, *P* < 0.001): ID increased from 0.7 ± 0.3 mm at 10 g to 6.9 ± 0.1 mm at 80 g, a strong linear correlation between CF and MSD, from 6.4 ± 0.7 mm at 20 g to 16.7 ± 0.1 mm at 80 g (*R*^2^ = 0.92, *P* < 0.001), and between CF and mSD, from 4.0 ± 0.4 mm at 20 g to 10.3 ± 0.0 mm at 80 g (*R*^2^ = 0.93, *P* < 0.001).

### Computer results

**Fig. 6:**
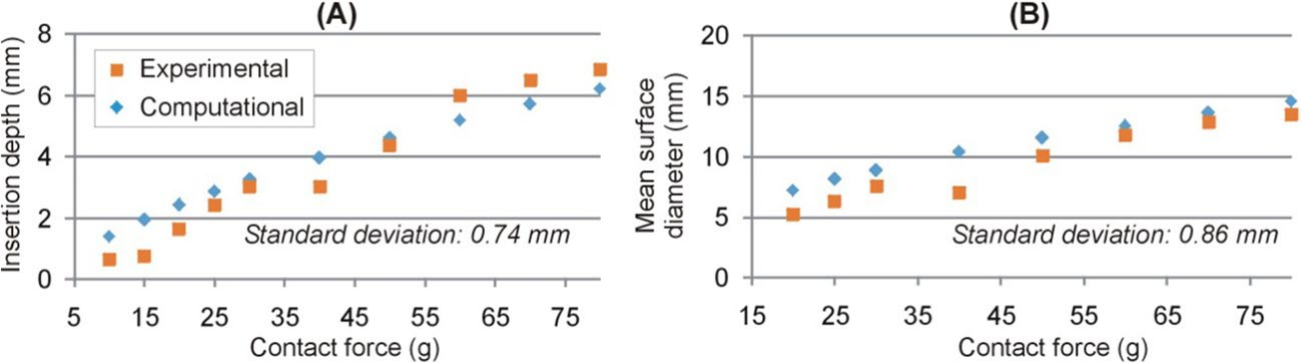
Comparison of experimental and computational results in terms of insertion depth (**A**) and mean surface diameter (**B**). The experimental results given in (**B**) are the mean values of the major and minor diameters given in Fig. 5

On testing different combinations for the Mooney-Rivlin model parameters, an optimal fit was achieved after 5 iterations, with an MSE less than 0.55 mm^2^ for ID. The model parameters were as follows: *c*_*10*_ = 1271, *c*_*01*_ = 1156, *c*_*11*_ = 1501, and *D* = 1.19 mm. Using these same values, the prediction of the diameter of the surface deformation offered an MSE of less than 0.65 mm^2^. The computational model results confirmed and emphasized the relationship between the mechanical deformation and the CF applied by the catheter tip on the cardiac muscle. As can be seen in Fig. 6, the computational model results agreed well with the experimental results in terms of both ID and the average value of MSD and mSD (note that the computational model had axial symmetry so that only one surface deformation diameter value can be computed). The standard deviation of the computational and experimental ID values and the mean surface diameter were 0.74 and 0.86 mm, respectively.

## Discussion

Cardiac ablation means that the metal catheter tip rests on the myocardium, inducing mechanical deformation on the tissue surface. Knowing the relationship between this deformation and the CF measured by the sensor at the catheter tip is thus crucial, since the effective tissue-electrode contact area largely determines the lesion size in RFCA [10, 11], and in turn the effectiveness of the ablation procedure. In fact, an increasing number of RFCA computational modeling studies incorporate the mechanical deformation problem to describe the relationship between deformation and CF [14–17]. It should thus be noted that this information is also important in the context of other cardiac catheter ablation methods, such as pulsed field ablation (PFA), that use high-voltage pulses to create irreversible pores in the cell membrane and induce death [20, 21]. In a previous modeling study, Pérez et al. proposed a mechanical model coupled with an electrothermal model to study RFCA, including the effect of heartbeat-induced electrode displacement [22], in which we considered a hyper-elastic Neo-Hookean model and CF values between 10 and 30 g. As in previous studies, the experiments were validated in terms of RF-induced lesion size, and not those of mechanical surface deformation. In contrast, in the present study, we considered a broader range of CF values of up to 80 g which implies larger deformations. For this reason, we opted for a Mooney-Rivlin instead of a Neo-Hookean model. While the latter is the simplest of all the hyper-elastic options in mathematical terms, it is limited to a relatively small strain (< 30%).

### Main findings

Although CF is known to play an important role and is related to lesion size [23], our experimental results provided all the relationships between the CF, surface deformation, and insertion depth, while the computer results confirmed that the Mooney-Rivlin model is suitable to study the mechanical deformation on ex vivo cardiac tissue samples due to exerting forces of up to 80 g through the catheter tip, i.e. the standard deviations of the computational and experimental ID values and mean surface diameter were of 0.74 and 0.86 mm, respectively. As expected, the mechanical deformation (assessed in terms of ID, MSD, and mSD) rose with increasing CF and showed strong correlations (*R*^2^ ≥ 0.92). The computer model can thus be considered useful for any future research study on minimally invasive cardiac procedures based on a catheter placed on the myocardium.

Interestingly, the deformed area was not symmetrical, i.e., not circular but elliptical, with two transversal diameters (major and minor). Although we did not use any method to accurately quantify the fiber orientation, the myocardium surface shape (see, e.g., Fig. 3A) suggested that the major surface diameter (MSD) followed that direction and its values ranged from 6.4 ± 0.7 mm at 20 g to 16.7 ± 0.1 mm at 80 g, while those of the mSD ranged from 4.0 ± 0.4 mm at 20 g to 10.3 ± 0.0 mm at 80 g, thus suggesting anisotropy around 60 to 80% (see Table 1). Like its electrical properties [24], the tissue’s mechanical properties are known to depend on the fiber orientation [25], so that it is difficult to assess the real impact of this anisotropy on the size and geometry of the RF-induced thermal lesion. Future computational modeling studies should be carried out to quantify this effect. If its importance were to be confirmed, our findings suggest that predictive models should consider the actual orientation of the fibers around the ablation zone, as has been done recently in terms of the electrical effect during cardiac ablation [26, 27].

**Table 1:** Relationship between mean values of major (MSD) and minor (mSD) diameters of surface deformation for different values of contact force (CF)

CF (g)	20	25	30	40	50	60	70	80
MSD (mm)	6.44	8.11	9.88	9.05	12.53	14.87	16.10	16.71
mSD (mm)	3.97	4.50	5.28	4.96	7.59	8.67	9.54	10.3
MSD/mSD (%)	**62.1**	**80.0**	**87.2**	**82.2**	**65.0**	**71.6**	**68.8**	**62.1**

**Fig. 7:**
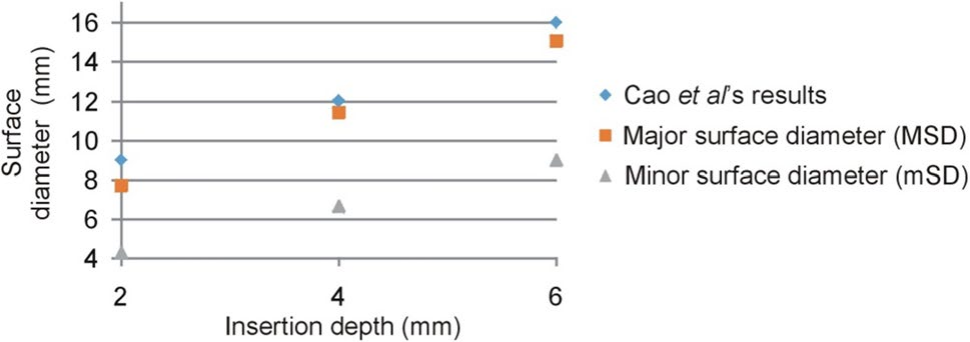
Comparison of the Cao et al. experimental results [12] and ours in terms of the relationship between insertion depth and surface deformation diameters

Despite the lack of previous results for the relationship between CF, ID, and surface deformation, we tried to partially compare our results to those reported in the literature [12, 13]. Cao et al. [12] characterized the deformation contour at different electrode penetration depths: 0 mm (no penetration), 2, 4, and 6 mm. They reported the deformation’s lateral extension, which was equivalent to the surface diameter in of our study. We then took their ID values (2, 4, and 6 mm) to estimate the equivalent CF value according to our fitting curve (see Fig. 5A), ID (mm) = 0.0942·CF − 0.2522, and we obtained the values of 24, 45, and 66 g, respectively. Finally, we compared the MSD and mSD values of surface deformation obtained from our experiments to those reported by Cao et al. [12]. As can be seen in Fig. 7, the comparison showed a satisfactory result in terms of MSD and a bias about 2.5 mm in terms of mSD. The differences could be explained by (1) the tissue type employed (bovine heart vs. porcine heart in ours), (2) the differences in the electrode design (2.6 mm rounded tip electrode vs. 2.33 mm flat-tip in ours), and (3) their results did not report any anisotropy since the *X*-ray system used to assess the deformation contour only took images on one plane.

**Fig. 8:**
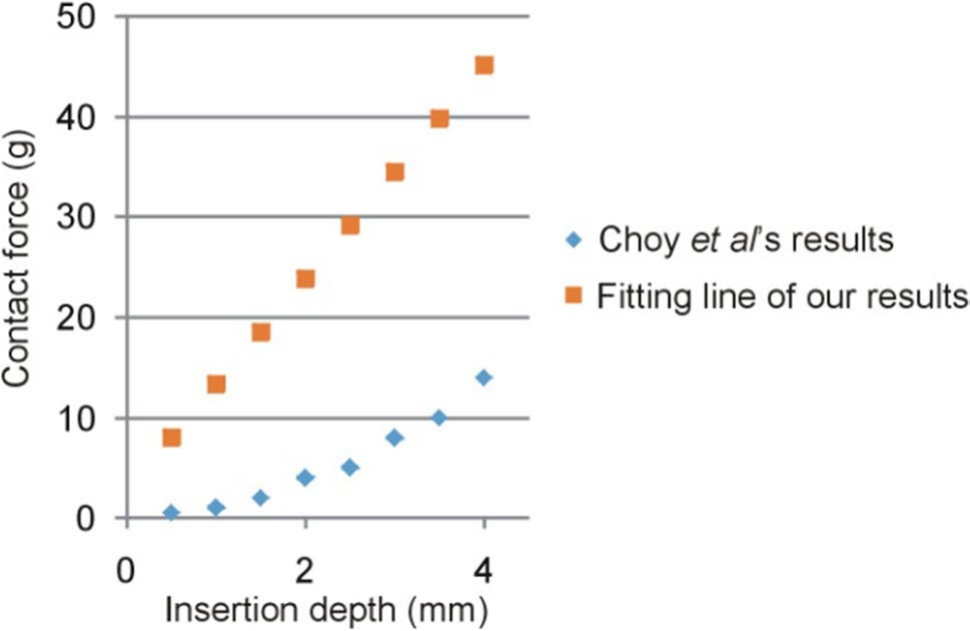
Comparison of the experimental results by Choy et al. [13] (taken at the left free cardiac wall 18 h after sacrifice) and ours in terms of the relationship between insertion depth and contact force. Our results are plotted as the line of best fit shown in Fig. 5A

Choy et al. [13] studied the relationship between the CF and ID by distinguishing the elastic and viscous behavior. They took physical measurements at different times after death and in three different endocardium areas. We selected only those taken at 18 h after sacrifice on the left ventricular free wall (the others obtained on the valve annuli) to compare their results with ours. Figure 8 shows Choy et al.’s results compared to our line of best fit (see Fig. 5A). The disagreement is evident, since while they only measured a CF of 14 g, our results suggest values of the order of 45 g for the same ID (e.g., 4 mm). This could be partially explained by the fact that their measurements were taken 60 s after each additional penetration depth was applied, which implies that they represents elastic responses (stiffness), i.e., that did not change with time. In fact, they found relaxation, i.e., reductions of around 30% in CF in the first 60 s, since the electrode was inserted at a given ID value, which is related to the tissue’s viscous behavior. We found even larger reductions, around 50% for the same period (data not formally presented since we were not interested in the viscous but only in the elastic behavior—stiffness). Unlike Choy et al.’s results, our reasonable agreement with those of Cao et al.’s was possibly due to they ignored the CF values once the insertion depth was set. In fact, we really inserted the catheter metal tip until reaching the CF target value and ignored the subsequent drop.

### Limitations

**Fig. 9:**
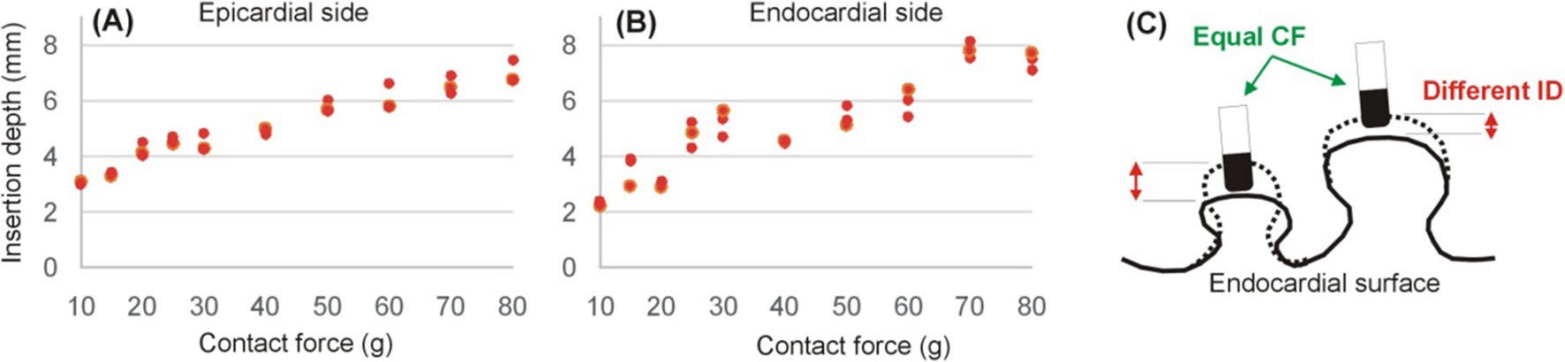
Relationships between insertion depth (ID) and contact force (CF) applied on epicardial (**A**) and endocardial (**B**) side. The greater dispersion of the results in endocardial applications is possibly due to the absence of a sufficiently flat area, which means that for equal values of CF, the insertion depth is varies widely according to the force distribution across the volume of the irregularities. Irregularities with a small volume tend to deform more easily and allow deeper insertions, as illustrated in panel (**C**)

The authors recognize the following limitations: First, the study was based on ex vivo porcine samples obtained within 24 h after sacrifice. The results would certainly be different in the case of a beating human cardiac wall. The computer model proposed here is thus at least valid for ex vivo experiments and is the first step towards developing a more realistic mechanical model able to capture the behavior of the in vivo cardiac wall. Secondly, the relaxation phenomenon associated with the tissue’s viscous behavior was ignored by taking the measurements almost immediately after pressing the catheter metal tip against the tissue surface. Thirdly, we used the epicardial side of the heart to conduct the experiments, even though most ablations involve an endocardial approach. This was because it is difficult to find a flat surface on the endocardial side due to the presence of papillary muscles and trabeculation. As regards this issue, we conducted a complementary set of experiments just for illustrative purpose in which the ID was only measured for different CF values (without the plastic plate used to facilitate the border of the surface deformation), comparing the endocardial and epicardial side (see Fig. 9). We found a markedly greater dispersion of the results in the endocardial case, since the insertion depth was much greater when applied on small-volume muscular structures (see Fig. 9C). Fourthly, the CF was measured using a force gauge firmly connected to the metal catheter tip so that only the axial force was assessed. This should be taken into account, due to some of the commercially available ablation catheters measuring the contact force in different directions and not only in that of the catheter axis. Fifthly, as mentioned in Section 2.4, the presence of the plastic plate and the pressure exerted on the tissue surface certainly has a mechanical impact on the experimental results. This plate is not present during RFCA and was included in the experiments to clearly identify the contours of the surface deformation. The plate was really included in the computer model (as shown in Fig. 4B) since we wanted to validate it experimentally. In this regard, there is no physical reason to suspect that the proposed computer model will not be valid to simulate cases without the plastic plate. Sixthly, our study only considered an electrode perpendicular to the tissue surface, even though we are aware that this position cannot always be used in clinical practice. This allowed us to significantly simplify the computational model by considering an axis of rotational symmetry around the catheter axis. Since our previous experimental study demonstrated that the catheter position has an impact on the electrode-tissue contact area [9], it is reasonable to assume that it will also impact the mechanical deformation profile of the tissue under the electrode. Despite this limitation, there are no physical reasons to suspect that the proposed model cannot be used as the basis to build a 3D model that considers different tissue/catheter angles. And finally, our mechanical model did not consider the anisotropy caused by the fiber orientation as analytically modeled in [28, 29], which could be included to achieve a better fit between the experimental and computational results in terms of different (major and minor) surface deformation diameters.

## Conclusions

In this paper, we propose a validated mechanical model for use in future contact-force guided RFCA models. This model presents for the first time a complete description of the relationships between the contact force, insertion depth, and surface deformation of cardiac tissue subjected to pressure by a perpendicularly placed ablation catheter. The experimental results obtained from an ex vivo porcine heart model were used to develop and validate a computational model, which used the Mooney-Rivlin model to reproduce the hyper-elastic behavior of the cardiac wall subjected to large deformations induced by forces of up to 80 g. In addition to the indirect clinical application through the improvement of computational models, our results confirm the linear relationship between contact force and insertion depth and also show that this correlation is lower when the electrode is resting on the endocardial side, possibly because the surface is not so flat as in the epicardium and presents irregularities marked by the presence of trabeculae. This should be considered in clinical practice for contact-force guided ablations, while its implication in terms of RF lesion size is a pending subject that we plan to address in future work.

## Supplementary Information

Below is the link to the electronic supplementary material.

Supplementary file1 (XLSX 58 KB)
